# Curated protein information in the *Saccharomyces* genome database

**DOI:** 10.1093/database/bax011

**Published:** 2017-03-11

**Authors:** Sage T. Hellerstedt, Robert S. Nash, Shuai Weng, Kelley M. Paskov, Edith D. Wong, Kalpana Karra, Stacia R. Engel, J. Michael Cherry

**Affiliations:** Department of Genetics, Stanford University, Stanford, CA 94305, USA

## Abstract

Due to recent advancements in the production of experimental proteomic data, the *Saccharomyces* genome database (SGD; www.yeastgenome.org) has been expanding our protein curation activities to make new data types available to our users. Because of broad interest in post-translational modifications (PTM) and their importance to protein function and regulation, we have recently started incorporating expertly curated PTM information on individual protein pages. Here we also present the inclusion of new abundance and protein half-life data obtained from high-throughput proteome studies. These new data types have been included with the aim to facilitate cellular biology research.

**Database URL**: www.yeastgenome.org

## Introduction

**Figure 1.**
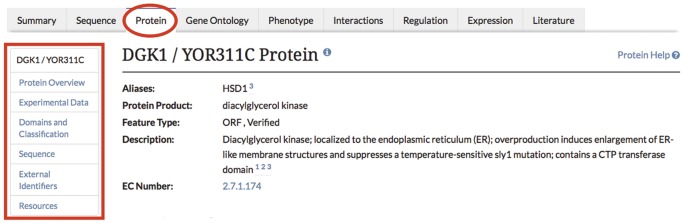
Descriptive information is included in the Overview section of protein pages in the SGD.

The *Saccharomyces* genome database (SGD; www.yeastgenome.org) is the premier community resource for curated data about the model organism *Saccharomyces cerevisiae* (1). As part of the SGD project we curate experimental protein results, with basic information summarized on the Locus Summary pages, and more detailed information presented on individual protein pages (2). The protein information housed at SGD can be used to direct experimental research aimed at elucidating protein function and biological role in the context of the cell. Currently, protein pages contain a descriptive overview of the protein in question (Figure 1), experimental data such as protein abundance and protein half-life, structural domain information, primary amino acid sequence from a variety of strains with overlaid experimental post-translational modification (PTM) data, physico-chemical properties derived from the protein sequence, a list of external identifiers and links to other resources that may be useful to researchers (Table 1).

**Table 1.** Various types of protein information are currently available on protein pages in the SGD

General information	Nomenclature Description EC Number
Experimental data	Protein abundance Protein half-life
Domains and classification	Computationally identified domains Domain locations Shared domain network visualization
Sequence	Protein sequence PTMs Sequence based physico-chemical properties (S288C)
External IDs	Cross-references to external databases
Resources	Homologs Protein databases Localization PTMs

As part of our continued effort to aid scientific discovery, we have expanded the types of information we collect during protein curation. Here we focus on the inclusion of PTM data in SGD, as well as new data on protein half-life and protein abundance. Finally, we discuss future directions aimed at enriching the experimental results integrated into SGD by expanding associated metadata and improved methods for visualization.

## PTM data

PTMs are critical to understanding mature protein function and regulation (3, 4). To enhance the community’s use of protein information, we have been actively curating experimentally determined PTMs since August 2014, with new knowledge added to SGD on a monthly basis.

### The curation strategy

**Figure 2.**
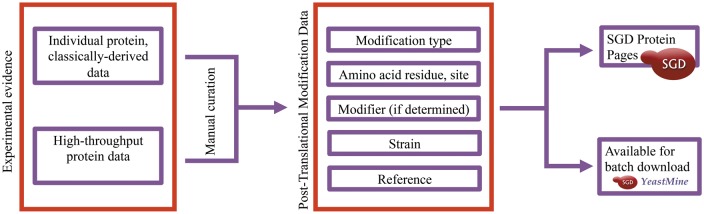
Integration of PTM information in the SGD is a multi-step process involving the identification of relevant papers, expert curation of PTM studies, and the visualization and display of PTM data.

Curation of PTM data is a multi-step process involving the identification of relevant articles, expert curation of PTM studies, and the visualization and display of PTM data (Figure 2).

*Identification of articles with PTM data.* As new articles are added to SGD, biocurators identify whether these papers contain experimentally derived PTM data and if so, they flag the article for data extraction. Relevant articles may contain high-throughput measurements, classically derived experimental results on individual proteins, or both.

*Expert curation of PTM studies.* After an article is identified as having possible PTM data, we extract the following types of information: proteins that are modified, amino acid residues that are modified, types of modifications, the modifier (protein performing the modification (if identified), such as the kinase responsible for a phosphorylation event), the strain background and the reference in which the modification is reported. The types of modifications currently curated at SGD as well as the number of annotations are shown in Table 2.

**Table 2.** Various types of PTMs have been integrated into the SGD

Modification type	Associated annotations (as of 19 October 2016)
phosphorylation	34 188
ubiquitination	6230
succinylation	1344
acetylation	925
methylation	284
palmitoylation	28
sumoylation	26
deacetylation	19
carbamidomethylation	16
dephosphorylation	10
butyrylation	10
ethylation	10

To extract this information, we identify the experimentally determined PTM data by examining the body of the text, the figures and often the supplementary material. In cases of high-throughput mass spectrometry experiments, the data are sometimes displayed as a peptide sequence with modified residues denoted by an asterisks or lowercase notation (5; e.g. K.MSphosFSGYSPKPI.S). In these instances, SGD’s Pattern Matching tool (www.yeastgenome.org/patmatch; 6) is utilized to determine and confirm the identity and position of the amino acid in the protein sequence that is modified, and in some cases, identify the protein itself. If there is any ambiguity in the residue identified, such as if the residue identified in the high-throughput analysis does not align with the residue in the protein sequence, the data point is not loaded. However, in the case of histone modification the modified residue at position *n* in the protein sequence is often reported as *n*−1 relative to the annotated chromosomal reference sequence defined by SGD due to the cleavage of the N-terminal methionine at the start site. These data are loaded after the residue position is adjusted.

**Figure 3.**
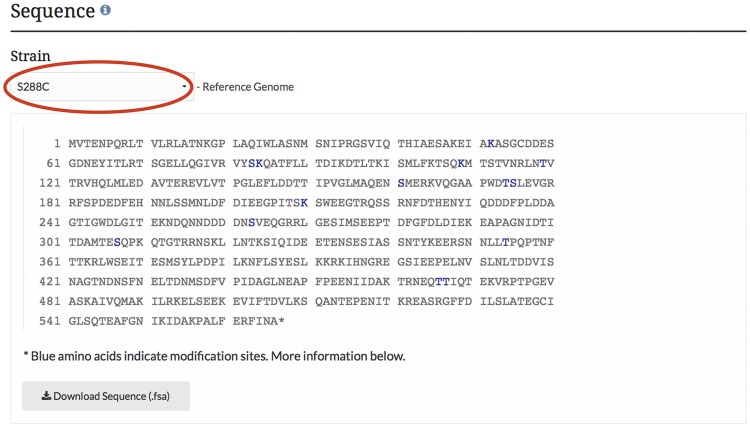
PTM information is integrated into the SGD and displayed on protein pages as highlighted residues overlaid on the protein sequence. The example shown here is from the *MCD1* protein page.

**Figure 4.**
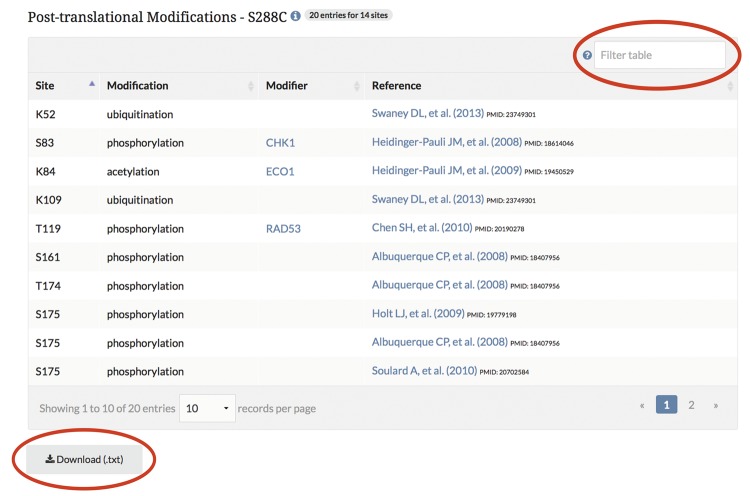
PTM information is integrated into the SGD and displayed on protein pages in a searchable, sortable table. The example shown here is from the *MCD1* protein page.

*Display and visualization on protein pages.* After curating the PTM information, it is integrated into the database and displayed on the protein pages as highlighted residues overlaid on the protein sequence (Figure 3), and in a searchable, sortable table (Figure 4).

As with many of the tools at SGD, users can change the sequence displayed to their yeast strain of choice by using the pull-down menu listing the 12 curated genome sequences maintained at SGD (7, 8). This pull-down feature allows visualization of amino acid changes across the protein sequences of the curated strains. For proteins in which sequence variation exists between strains, the display of modified residues will change if protein sequence differences exist at modification sites. By changing the strain in the pull-down menu, one can explore PTM variations between the strains, as the curated data change in the protein sequence as well as in the modification table. Data in this table can be sorted and/or filtered based on the site, modification, modifier, or curated reference. The data in the table are also available for download as a text (.txt) file. Curated data for the entire proteome can be retrieved and downloaded as a tab-separated values (.tsv) file using YeastMine (yeastmine.yeastgenome.org; 9), SGD’s instance of the InterMine search and retrieval tool (see below).

### Protein abundance and half-life data

**Figure 5.**
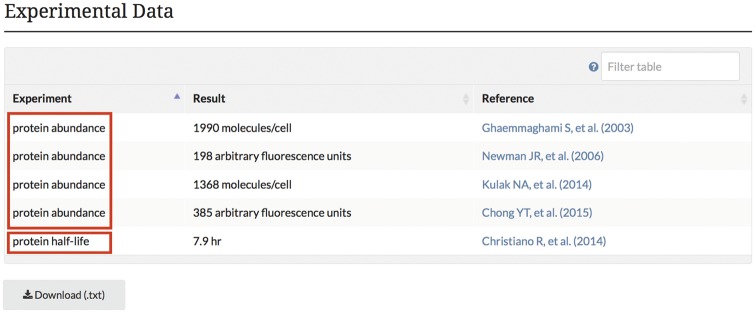
Experimental abundance and half-life data obtained from high-throughput proteome studies are integrated into the SGD and displayed on protein pages in a searchable, sortable table. The example shown here is from the *STH1* protein page.

In addition to these new advancements in the presentation of protein modification data, SGD protein pages now display new abundance and half-life data obtained from high-throughput proteome studies (10–14). These data are displayed on the protein pages in a searchable, downloadable table like many tables found at SGD (Figure 5). These data are also available through YeastMine, and include the type of experiment (abundance or protein half-life), associated units, and corresponding publication.

### Finding protein information in YeastMine

**Figure 6.**
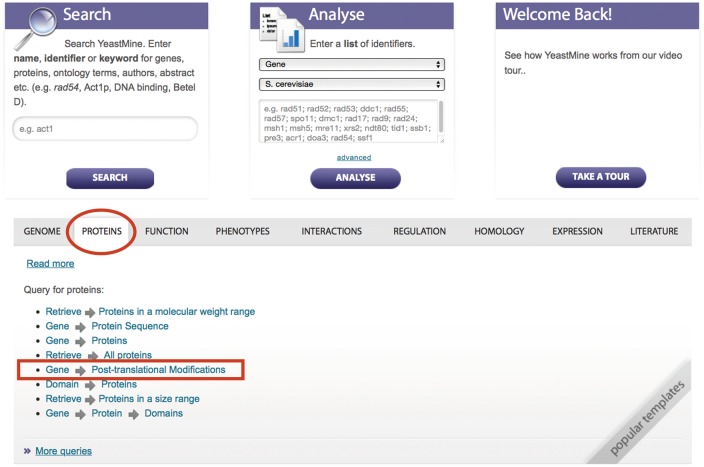
PTM information can be queried and retrieved using the ‘Gene → PTM’ template in YeastMine (yeastmine.yeastgenome.org).

YeastMine is a powerful, multifaceted search and data retrieval tool powered by InterMine that contains all the manual, high-throughput, and computational data available in SGD (15). YeastMine serves as a data warehouse, presenting researchers with a simple means to access data for proteins of interest. A large number of pre-defined query templates are available in YeastMine, as well as many lists of features in the database. Curated PTM data are one of the many types of data integrated into YeastMine. By using existing templates and providing the opportunity to create tailored lists, users can easily access some or all of the PTM and protein abundance data. PTM data can be queried and retrieved using the ‘Gene → PTM’ template located in the “template” section within the ‘protein’ category filter (Figure 6).

In addition to PTM data, protein abundance data can be accessed using one of two templates. ‘Gene → Protein Abundance’ facilitates the retrieval of user-defined abundance data for a single protein all the way up to the entire proteome. A second template ‘Retrieve → Proteins in a given abundance range’ permits users to retrieve a list of genes encoding proteins within a user specified abundance range by defining the upper and lower end of the range. Once retrieved, these data can be exported, or used to create lists for further analysis within YeastMine. Two new templates were recently created to provide users with access to protein half-life information obtained from a study by Christiano *et al.*, 2014 (14). The ‘Gene → Protein Half-life’ template facilitates the retrieval of half-life information of the protein for a specified gene or lists of genes. The ‘Retrieve → Proteins with half-life in a given range’, template retrieves a list of genes encoding proteins within a user defined range.

### Future directions

The increase in the publication of protein experimental data has driven SGD to expand the types of protein annotations we provide in order to better facilitate the advancement of cellular biology research. We will continue to provide users with newly curated modification data, including the experimental and cellular conditions under which the data were generated, as well as protein complexes acting as modifiers. As more curated data are added to SGD, future advancements will include more explicit differentiation between types of PTMs, such as with colour changes, or icons, to allow for better visualization. We also plan to overlay PTM data on SGD’s Variant Viewer sequence alignment tool, which displays sequence variants and similarity scores for open reading frames (ORFs) within SGD’s reference genome panel of 12 widely used *S. cerevisiae* strains (www.yeastgenome.org/variant-viewer; 16). Combining PTM data with Variant Viewer will facilitate mapping of sequence polymorphism with modification sites.

Further advancements will also be made to the protein abundance and half-life data to record experimental methods, conditions, and effectors, in order to aid users in understanding the differences between experimental values. As more studies on protein abundance and half-life are published, we will continue to add high-quality datasets to SGD to enrich the data we currently make available. In addition, we will work to incorporate enhanced data visualization methods to better distinguish different datatypes and provide contextual, as well as baseline, information. We encourage user feedback regarding enhancements of SGD’s currently available data, tools, and visualizations.

These data will be presented in different ways to suit different needs. Although tabular format whether on SGD protein pages or in YeastMine, is good for presenting complete details if the amount of information is small, these tables are generally quite large for well-studied proteins, making it a challenge for users to consume the available knowledge and synthesize it into a coherent biological story. Therefore, we will introduce protein summaries written in plain language in order to help people understand and serve the broadest possible audience. Other curated data types in SGD, such as function, phenotype and regulation data, already have written summaries on gene pages. Although there is a large amount of biological research and experimental data available for *S. cerevisiae*, and tens of thousands of annotations already exist in the comprehensively annotated SGD, there remain hundreds of proteins whose function is still unknown. The careful assimilation and contextualization of expert knowledge we provide through our protein curation efforts support students, educators, and scientists who further utilize the information downstream in many ways on a daily basis in the course of scientific discovery.

## Funding

This work was supported by a grant from the National Human Genome Research Institute at the US National Institutes of Health to the SGD project (U41 HG001315). The content is solely the responsibility of the authors and does not necessarily represent the official views of the National Human Genome Research Institute or the National Institutes of Health. 

*Conflict of interest*. None declared.
